# QTL mapping of yield component traits on bin map generated from resequencing a RIL population of foxtail millet (*Setaria italica*)

**DOI:** 10.1186/s12864-020-6553-9

**Published:** 2020-02-10

**Authors:** Tianpeng Liu, Jihong He, Kongjun Dong, Xuewen Wang, Wenwen Wang, Peng Yang, Ruiyu Ren, Lei Zhang, Zhengsheng Zhang, Tianyu Yang

**Affiliations:** 1 Crop Research Institute, Gansu Academy of Agricultural Sciences, Lanzhou, 730070 Gansu People’s Republic of China; 2 College of Agronomy and Biotechnology, Southwest University, Chongqing, 400716 People’s Republic of China; 3 Department of Genetics, University of Georgia, 120 Green Str, Athens, GA 30602 USA

**Keywords:** Foxtail millet (*Setaria italica*), Yield component traits, SNP, Bin map, QTL

## Abstract

**Background:**

Foxtail millet (*Setaria italica*) has been developed into a model genetical system for deciphering architectural evolution, C_4_ photosynthesis, nutritional properties, abiotic tolerance and bioenergy in cereal grasses because of its advantageous characters with the small genome size, self-fertilization, short growing cycle, small growth stature, efficient genetic transformation and abundant diverse germplasm resources. Therefore, excavating QTLs of yield component traits, which are closely related to aspects mentioned above, will further facilitate genetic research in foxtail millet and close cereal species.

**Results:**

Here, 164 Recombinant inbreed lines from a cross between Longgu7 and Yugu1 were created and 1,047,978 SNPs were identified between both parents via resequencing. A total of 3413 bin markers developed from SNPs were used to construct a binary map, containing 3963 recombinant breakpoints and totaling 1222.26 cM with an average distance of 0.36 cM between adjacent markers. Forty-seven QTLs were identified for four traits of straw weight, panicle weight, grain weight per plant and 1000-grain weight. These QTLs explained 5.5–14.7% of phenotypic variance. Thirty-nine favorable QTL alleles were found to inherit from Yugu1. Three stable QTLs were detected in multi-environments, and nine QTL clusters were identified on Chromosome 3, 6, 7 and 9.

**Conclusions:**

A high-density genetic map with 3413 bin markers was constructed and three stable QTLs and 9 QTL clusters for yield component traits were identified. The results laid a powerful foundation for fine mapping, identifying candidate genes, elaborating molecular mechanisms and application in foxtail millet breeding programs by marker-assisted selection.

## Background

Foxtail millet (*S. italica*), a diploid species (2n = 2x = 18) domesticated from its wild relative green millet (*Setaria viridis*) with A genome of the *Setaria* [1, 2], is mainly cultivated in China, India, Japan and some arid and semi-arid regions as a stable food grain. In addition, it is also used as a forage crop in North America, Africa and Australia [2, 3]. Due to a small genome size, self-fertilization, short growing cycle, small growth stature, efficient genetic transformation and abundant diverse germplasm resources [4–6], *S. italica* and *S. viridis* have been developed into model genetic systems for deciphering architectural evolution, C_4_ photosynthesis, nutritional properties, abiotic tolerance and bioenergy in cereal grasses [7–10]. Straw weight per plant (SWP), panicle weight per plant (PWP), grain weight per plant (GWP) and 1000-grain weight (TGW) are the most important traits to foxtail millet as a food and forage crop or model genetic system and closely related with agricultural production. However, compared to other starch cereal crops, few studies were carried out for QTLs of yield component traits in *Setaria* [11].

The release of *S. italica* genome sequence in 2012 [12, 13] has greatly facilitated large-scale development of genomic resources. Pandey et al. [14], Zhang et al. [15] and Fang et al. [16] scanned the whole genome sequence of foxtail millet and developed 28,342, 5020 and 10,598 simple sequence repeat (SSRs) makers, respectively, that were used to construct genetic or physical map for foxtail millet. Simultaneously, researchers applied different segregating populations to map various agro-morphological traits. Doust et al. [17] used F_2_ interspecies population from a cross between *S. italica* accession B100 and *S. viridis* accession A10 to locate 25 QTLs for vegetative branching and inflorescence architecture. Mauro-Herrera et al. [18] identified 16 flowering time QTLs in B100 × A10 F_7_ RILs. Using F_2:3_ and RIL populations generated from the B100 × A10 cross, Odonkor et al. [19] identified the presence of an additive main effect QTL for reduced shattering on chromosomes V and IX. Moreover, Wang et al. [20] detected five QTLs closely related to plant morphological traits and grain weight using a Shen3 × Jinggu20 F_2_ intraspecific population. Sato et al. [21] mapped a responsible gene *stb1* on chromosome 2 by two F_2_ intraspecies populations. Fang et al. [16] identified 29 QTLs for 11 agronomic and yield traits applying a Longgu7 × Yugu1 F_2_ intraspecific population. Gupta et al. [22] identified eight SSR markers on different chromosomes showing significant associations with nine agronomic traits in a natural population consisting of 184 foxtail millet accessions from diverse geographical locations.

With the availability of high-throughput genotyping technology, the rapid investigation of genomic variation in both natural populations and segregating populations of foxtail millet is now feasible by genotyping using SNPs. Jia et al. [23] sequenced 916 diverse foxtail millet varieties and identified 2,584,083 SNPs and used 845,787 common SNPs to construct a haplotype map of the foxtail millet genome. Five hundred and twelve loci associated with 47 agronomic traits were identified through genome wide association studies (GWAS). Ni et al. [24] and Zhang et al. [25] resequenced a RIL population using single seed descent strategy from a cross between Zhanggu and A2, and developed a high-resolution bin map with high-density SNP markers. A total of 69 QTLs for 21 agronomic traits were identified. Wang et al. [26] mapped 11 major QTLs of eight agronomic traits using RAD-seq to detect SNP markers and screen F_2_ progenies derived from the cross between Hongmiaozhangu and Changnong35. In another study, Wang et al. [27] identified 57 QTLs related to 11 agronomic traits in an F_2_ mapping population from a cross between Aininghuang and Jingu21. These studies provided lots of information for genetic improvement and gene discovery.

In present study, we adopted high-throughput whole-genome resequencing to construct high-density bin map and focused on identifying QTLs of the yield component traits, which led to 47 QTLs including three stable QTLs. The results will be valuable for further research on fine mapping, identifying candidate genes, elaborating molecular mechanisms and marker-assisted selection (MAS) in foxtail millet.

## Results

### Phenotypic evaluation

All four yield component traits (Table 1) in Yugu1 were higher than those in Longgu7 under five tested environments from different agricultural areas in northwest China. Difference of yield component traits in the RIL population had a wide range and exhibited an obvious transgressive segregation in five environments. All traits were approximately prone to normal distribution via skewness and kurtosis tests, and the variance value of each trait was relatively large except that of TGW, which indicated that the RIL population was conducive to QTL mapping SWP, PWP and GWP which had great potentials for genetic improvement. Significant correlations were found among SWP, PWP and GWP (Table 2). However, correlation was inconsistent between TGW and other traits under five environments, indicating that the interactions between SWP, PWP, GWP and TGW were potentially influenced by environmental conditions. Moreover, analyses of variance indicated highly significant genotypic and environmental effects (*p < 0.01*) for all measured traits (Table 3), which suggested that environmental factors had great effect on foxtail millet yield component traits.

**Table 1:** Variation of yield component traits for Longgu7, Yugu1, and their RIL population

Trait	Environment	Parents			Population							
		P_1_	P_2_	P_1_- P_2_	Range	Min	Max	Mean	SD	Variance	Skewness	Kurtosis
SWP	2017-DH	9.08	15.54	− 6.46	16.90	5.41	22.31	11.59	3.27	10.71	0.63	0.22
	2017-HN	13.77	23.82	−10.05	24.82	9.66	34.48	20.12	4.87	23.75	0.20	−0.18
	2017-WW	9.37	17.61	−8.24	22.20	7.20	29.40	18.83	3.85	14.83	0.33	0.57
	2018-GG	12.35	25.45	−13.1	22.84	9.27	32.10	18.57	4.56	20.76	0.65	0.50
	2018-HN	16.87	27.15	−10.28	25.63	9.42	35.05	20.54	5.18	26.79	0.45	−0.12
PWE	2017-DH	12.35	16.91	−4.56	19.01	7.57	26.58	13.72	3.44	11.82	0.96	1.85
	2017-HN	11.83	21.19	−9.36	21.60	5.56	27.16	13.50	3.34	11.13	0.69	1.59
	2017-WW	10.64	11.81	−1.17	17.87	7.33	25.20	15.68	3.32	11.05	0.43	0.15
	2018-GG	12.12	19.14	−7.02	20.00	5.38	25.37	14.02	3.71	13.79	0.50	0.10
	2018-HN	16.94	34.37	− 17.43	29.70	9.65	39.34	23.36	4.88	23.82	0.36	0.59
GWP	2017-DH	8.86	13.25	−4.39	15.93	4.01	19.94	9.92	2.67	7.14	0.79	1.21
	2017-HN	9.97	16.71	−6.74	19.98	3.38	23.36	10.81	2.82	7.95	0.74	2.58
	2017-WW	8.17	9.36	−1.19	19.57	5.53	25.11	13.04	3.05	9.30	0.61	1.21
	2018-GG	10.15	12.52	−2.37	13.74	2.91	16.65	9.30	3.13	9.79	0.20	−0.63
	2018-HN	14.13	31.25	−17.12	26.78	7.50	34.29	19.80	4.04	16.30	0.31	0.90
TGW	2017-DH	2.64	2.92	−0.28	1.20	2.00	3.20	2.66	0.24	0.06	−0.08	0.01
	2017-HN	3.42	3.90	−0.48	1.45	2.54	3.99	3.25	0.24	0.06	0.07	0.56
	2017-WW	2.56	2.99	−0.43	1.30	2.40	3.70	2.86	0.23	0.05	0.54	0.64
	2018-GG	2.61	2.75	−0.14	2.43	1.54	3.97	2.36	0.33	0.11	0.69	2.23
	2018-HN	3.66	3.77	−0.11	1.80	2.40	4.20	3.39	0.30	0.09	0.19	0.40

*SWP* Straw weight per plant, *PWP* Panicle weight per plant, *GWP* Grain weight per plant, *TG*W 1000-grain weight. *DH* Dunhuang, *HN* Huining, *WW* Wuwei, *GG* Gangu. 2017 and 2018 represented years. P_1_:Longgu7; P_2_: Yugu1

**Table 2:** Correlation analysis among yield component traits under five environments

Environment	Traits	SWP	PWP	GWP	TGW
2017-DH	SWP	1.00			
	PWP	0.29^**^	1.00		
	GWP	0.28^**^	0.93^**^	1.00	
	TGW	0.27^**^	0.35^**^	0.36^**^	1.00
2017-HN	SWP	1.00			
	PWP	0.26^**^	1.00		
	GWP	0.18^*^	0.90^**^	1.00	
	TGW	0.10	0.25^**^	0.22^**^	1.00
2017-WW	SWP	1.00			
	PWP	0.53^**^	1.00		
	GWP	0.50^**^	0.90^**^	1.00	
	TGW	0.01	0.25^**^	0.22^**^	1.00
2018-GG	SWP	1.00			
	PWP	0.53^**^	1.00		
	GWP	0.36^*^	0.80^**^	1.00	
	TGW	0.12	0.37^**^	0.39^**^	1.00
2018-HN	SWP	1.00			
	PWP	0.36^**^	1.00		
	GWP	0.41^**^	0.93^**^	1.00	
	TGW	0.12	0.09	0.11	1.00

*, ** Significant differences with a probability level of 0.05 and 0.01, respectively. The statistical method Pearson correlation coefficient is used

**Table 3:** Analysis of univariate general linear model for yield related traits across five environments for the Longgu7 × Yugu1 RIL population

Trait	Factor	Sum of squares	DF	Mean Square	F
SWP	Environment	8604.91	4	2151.23	191.98^**^
	Genotype	8433.02	163	51.74	4.62^**^
	Error	7261.20	648	11.21	
PWP	Environment	11,286.77	4	2821.69	233.99^**^
	Genotype	3801.88	163	23.32	1.93^**^
	Error	7765.90	644	12.06	
GWP	Environment	11,853.99	4	2963.50	316.08^**^
	Genotype	2124.08	163	13.03	1.39^**^
	Error	6028.68	643	9.38	
TGW	Environment	111.72	4	27.93	530.97^**^
	Genotype	25.76	163	0.16	3.00^**^
	Error	33.19	631	0.05	

** Significant differences with a probability level of 0.01 with univariate general linear model analyses

### Sequencing and SNP identification

**Fig. 1:**
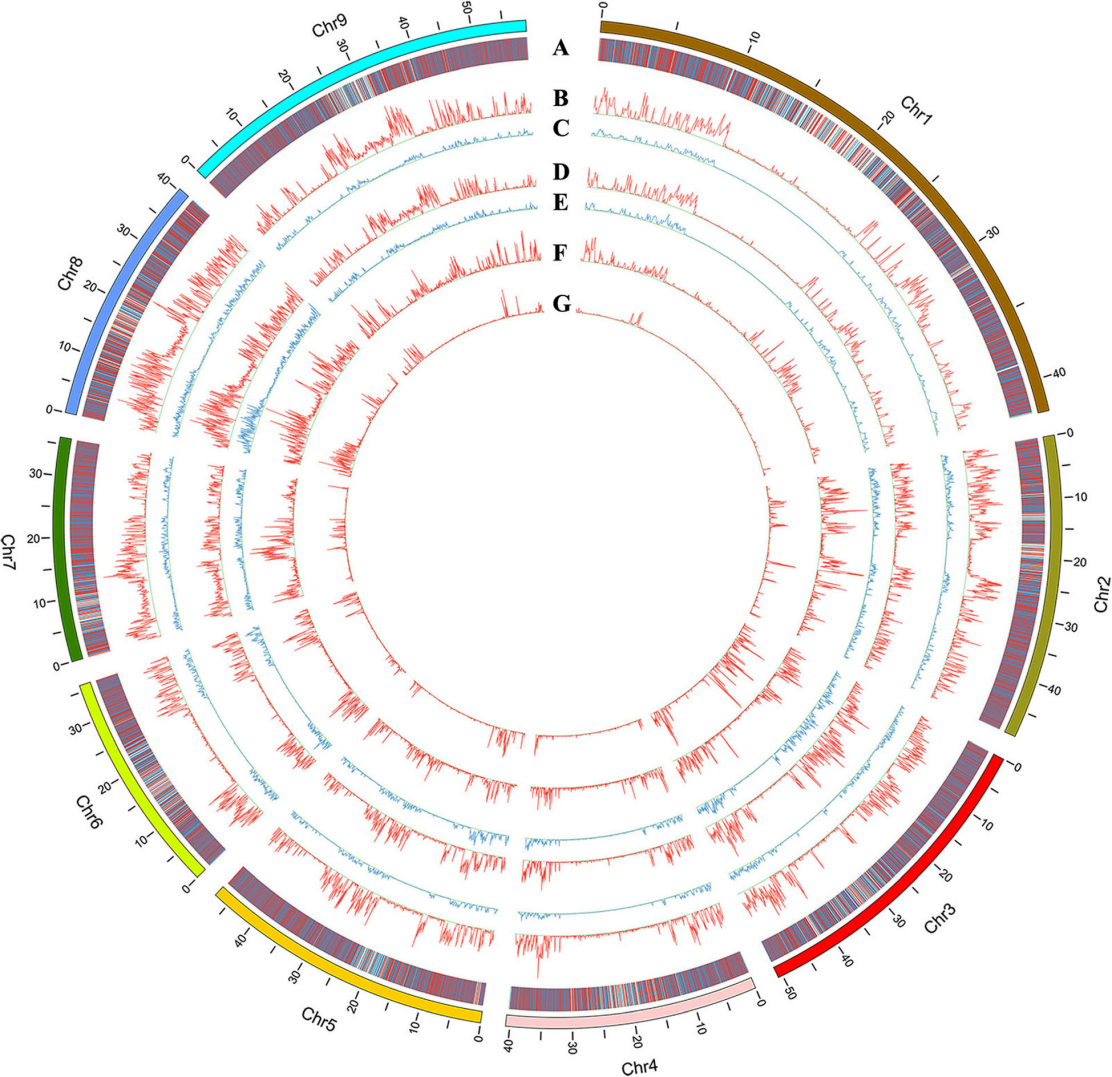
Genes, SNP, InDel and specific SNP distribution on chromosomes by the two parents aligned with the reference genome. **a**: Gene positions (red = forward; blue = reverse); **b**: SNPs per 50Kb on Longgu7 (max = 1647); **c**: InDels per 50Kb on Longgu7 (max = 122); **d**: SNPs per 50Kb on Yugu1 (max = 1490); **e**: InDels per 50Kb on Yugu1 (max = 122); **f**: SNPs exclusive from Longgu7 per 50Kb (max = 1198); **g**: SNPs exclusive from Yugu1 per 50Kb (max =1172)

We resequenced both parents with 20x depth and 164 RILs with 5x depth on an Illumina HiSeq platform and produced clean data for mining SNPs and developing bin markers. By aligning clean reads with the reference genome sequence of *Setaria italic*, we obtained 1,865,169 SNPs and 161,602 InDels in Longgu7, and 1,394,661 SNPs and 103,709 InDels in Yugu1. According to alignment between two parents, common SNPs were discarded (Additional file 1: Table S1). Finally, 759,243 and 288,735 parental specific SNPs were identified in Lugu7 and Yugu 1, respectively (Fig. 1, Additional file 1: Table S1). The number of SNPs on each chromosome ranged from 10,341 to 149,341 (Additional file 1: Table S1). We obtained 3413 bin markers by sliding window of 15 SNPs (Additional file 2: Table S2).

### Recombination breakpoint determination and genetic map construction

**Fig. 2:**
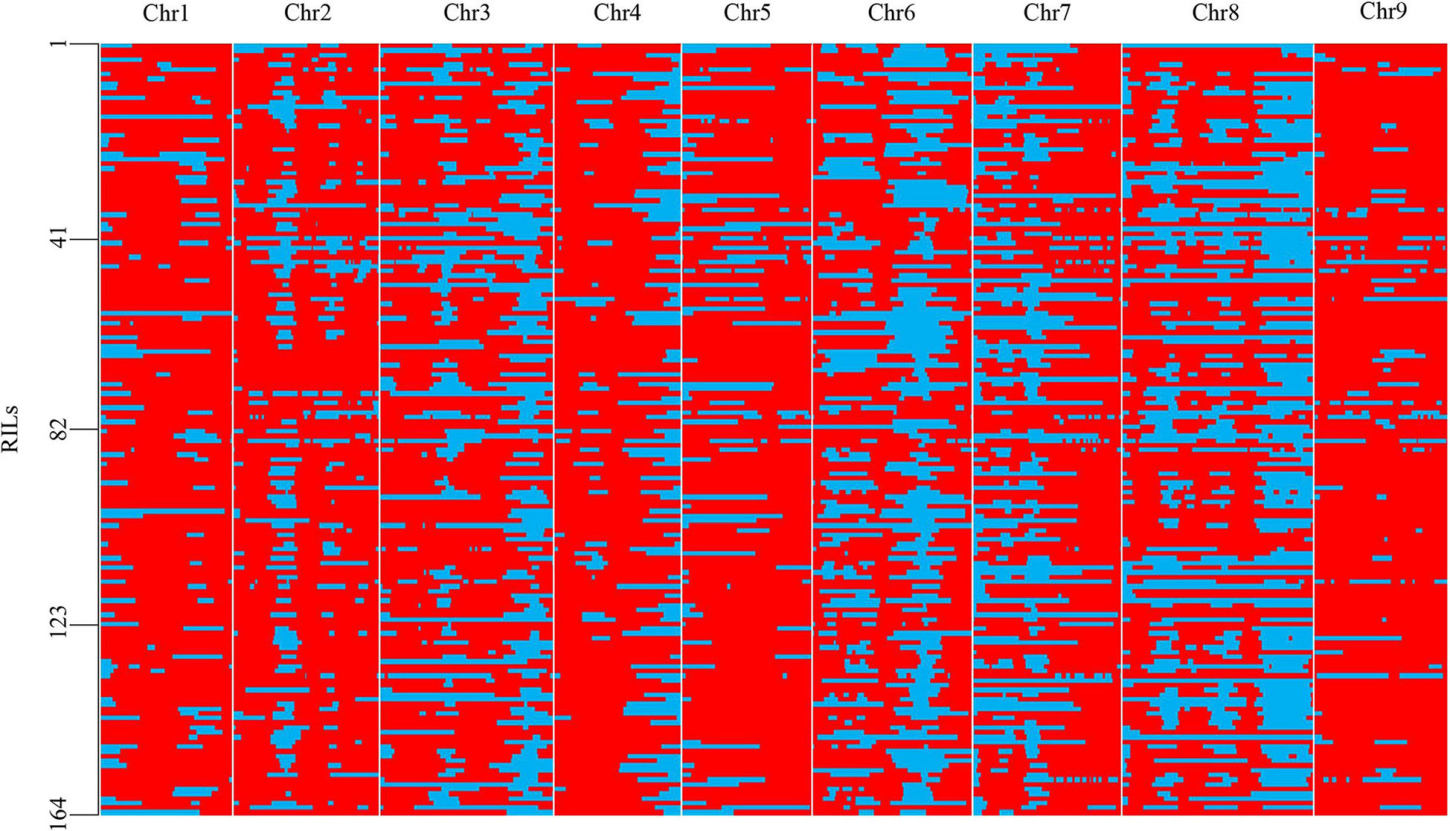
Recombination bin map of 164 foxtail millet RILs. The whole map contains 3413 bin markers and 3963 breakpoints. Red: genotype of Longgu7; blue: genotype of Yugu1. Left number represent the number of recombinant inbred lines. Chromosomes are separated by vertical white lines. Chr: chromosome; RIL: recombinant inbred line

The recombination breakpoints were checked by the bin positions where genotypes were changed from one type to the other along the chromosome. A total of 3963 breakpoints were identified among 164 RILs and the average of breakpoints per line was 24.16 (Additional file 3: Table S3, Additional file 4: Table S4). Then, these recombination breakpoints of 164 lines were used to construct a skeleton binmap (Fig. 2). The physical length of each bin ranged from 47.76 kb to 293.38 kb (Additional file 3: Table S3). These bins were regarded as genetic bin makers for the construction of the linkage map that spanned 1222.26 cM of the foxtail millet genome with 0.36 cM/bin. The average distance of adjacent bin markers ranged from 0.27 to 0.40 cM for all nine chromosomes (Additional file 3: Table S3, Additional file 7: Figure S1).

### Segregation distortion

Among the 3413 mapped bin markers, 2935 showed segregation distortion (*p* < *0.05*) (Additional file 8: Figure S2, Additional file 5: Table S5) accounting for 89.10% of the total. These 2935 bin markers comprised 31 segregation distortion regions (SDRs) which were unevenly distributed on nine chromosomes. All markers on Chr1, Chr5 and Chr9 exhibited segregation distortion and contained abrupt segregation distortion peaks. Two peaks were located between Bin0100 and Bin0175 on Chr1, one at Bin1447 on Chr5 and one on end of Chr9. Chr4 had two segregation distortion peaks on Bin1200 and Bin1249 at one SDR with 80.52% bin markers. Chr2 had two SDRs accounting for 89.10% bin markers. Chr6 and Chr7 carried five SDRs with 86.56 and 80.48% bin markers and obvious segregation distortion peaks on proximal end of chromosome. Chr3 had six SDRs with 86.40% bin markers. There were nine SDRs on Chr8, which included two identical SDRs harboring gametocidal genes at the middle-upper and bottom of Chromosome in previous report [16]. Three hundred and fifty of the 2935 (11.93%) bin markers attributed to Yugu1 alleles and the remaining bin markers (88.07%) favored Longgu7 alleles. Furthermore, recombinant fraction of markers on peaks of all SDRs was lower than other regions, which may be caused by the tighter linkage of chromosome fragment on SDRs (Additional file 8: Figure S2).

### QTL mapping of yield component traits

Forty-seven QTLs of yield component traits were identified under five environments and explained 5.5–14.7% of phenotypic variation. Among these QTLs, 39 favorable QTL alleles for yield component traits are originated from Yugu1 except qGWP2.1, qSWP 6.1, qSWP 6.2, qPWP6.2, qPWP6.3, qGWP6.1, qTGW6.1 and qSWP8.2 (Table 4).

**Table 4:** QTL identified for four yield component traits under multi-environments based on bin markers genetic map

Traits	QTL	Environment	Chromosome	Nearest locus	Location	LOD	Additive -effect	PVE (%)
SWP	qSWP1.1	2017-WW	1	Bin0060	17.19	2.40	−2.15	6.5
	qSWP1.2	2017-WW	1	Bin0179	47.89	2.13	−2.69	5.8
	qSWP2.1	2017-DH	2	Bin0525	133.99	2.05	−1.23	5.6
		2018-HN	2	Bin0525	133.99	2.78	−2.27	7.7
	qSWP3.1	2018-GG	3	Bin1095	202.88	2.50	−1.30	6.8
	qSWP3.2	2017-DH	3	Bin0601	16.58	2.19	−1.17	6.0
	qSWP6.1	2018-GG	6	Bin1554	23.12	2.83	1.26	7.6
	qSWP6.2	2017-HN	6	Bin1635	52.97	2.49	1.28	6.7
		2017-DH	6	Bin1632	52.05	3.87	1.07	10.4
	qSWP7.1	2017-HN	7	Bin2012	14.29	2.36	−1.24	6.4
		2017-WW	7	Bin2020	18.93	3.46	−1.22	9.3
	qSWP7.2	2017-WW	7	Bin2100	51.61	3.40	−1.34	9.1
	qSWP7.3	2017-HN	7	Bin2202	100.49	2.70	−1.45	7.3
	qSWP7.4	2018-GG	7	Bin2263	119.53	2.45	−1.69	6.7
		2017-WW	7	Bin2259	118.30	4.76	−1.96	12.5
		2018-HN	7	Bin2250	115.23	2.49	−1.68	6.9
	qSWP7.5	2017-HN	7	Bin2297	130.27	2.07	−1.70	5.7
	qSWP8.1	2018-GG	8	Bin2418	26.66	2.23	−1.38	6.1
		2018-HN	8	Bin2418	26.66	2.47	−1.65	6.8
	qSWP8.2	2017-WW	8	Bin2466	47.01	2.26	0.98	6.1
	qSWP8.3	2018-GG	8	Bin2538	83.65	3.25	−1.66	8.7
	qSWP9.1	2017-HN	9	Bin3320	28.54	3.92	−2,00	10.4
		2018-GG	9	Bin3309	25.16	3.24	−2.05	8.7
		2017-WW	9	Bin3304	23.63	3.19	−1.95	8.6
	qSWP9.2	2017-WW	9	Bin3343	35.90	5.67	−2.20	14.7
		2018-HN	9	Bin3367	42.61	2.57	−2.23	7.1
PWP	qPWP2.1	2018-HN	2	Bin0356	73.51	2.83	−2.21	7.7
	qPWP3.1	2018-GG	3	Bin0814	81.88	2.52	−1.22	6.8
	qPWP3.2	2018-GG	3	Bin0997	156.50	4.10	−1.22	10.9
		2018-HN	3	Bin0997	156.50	2.80	−1.35	7.6
	qPWP3.3	2018-GG	3	Bin1093	202.27	3.57	−1.24	9.6
		2018-HN	3	Bin1100	204.73	3.41	−1.60	9.2
	qPWP5.1	2018-HN	5	Bin1491	42.98	2.61	−2.18	7.1
	qPWP6.1	2018-GG	6	Bin1504	2.76	2.03	−1.67	5.5
	qPWP6.2	2017-DH	6	Bin1636	52.27	2.93	0.99	8.2
	qPWP6.3	2017-HN	6	Bin1806	116.63	3.32	1.20	8.9
		2017-WW	6	Bin1774	104.32	2.32	0.84	6.3
	qPWP7.1	2018-GG	7	Bin2359	148.38	2.98	−2.30	8.0
	qPWP7.2	2018-HN	7	Bin2202	100.50	4.05	−1.77	10.8
	qPWP8.1	2018-HN	8	Bin3046	275.59	2.45	−1.28	6.7
	qPWP9.1	2017-WW	9	Bin3222	2.76	3.05	−1.65	8.2
	qPWP9.2	2018-HN	9	Bin3281	16.57	2.53	−2.45	6.9
		2017-HN	9	Bin3294	20.25	2.74	−1.89	7.4
	qPWP9.3	2018-HN	9	Bin3406	53.87	3.43	−3.42	9.2
GWP	qGWP2.1	2017-HN	2	Bin0278	27.74	2.62	0.84	7.1
	qGWP2.2	2018-HN	2	Bin0356	73.51	3.67	−2.07	9.8
	qGWP3.1	2018-GG	3	Bin0621	17.19	2.54	−1.16	6.9
		2018-HN	3	Bin0632	19.34	2.20	−1.33	6.0
	qGWP3.2	2018-GG	3	Bin0814	81.88	2.43	−1.01	6.6
		2018-HN	3	Bin0793	76.66	2.13	−1.24	5.8
	qGWP3.3	2018-GG	3	Bin0994	153.05	2.89	−0.89	7.8
		2017-DH	3	Bin1004	161.48	2.20	−0.67	6.3
		2018-HN	3	Bin0997	156.50	2.93	−1.14	7.9
	qGWP6.1	2017-HN	6	Bin1806	116.63	4.22	1.15	11.2
		2018-HN	6	Bin1798	113.24	2.11	1.08	5.8
	qGWP7.1	2018-HN	7	Bin2196	99.27	2.74	−1.23	7.5
	qGWP7.2	2018-GG	7	Bin2359	148.38	2.18	−1.59	6.0
	qGWP8.1	2018-HN	8	Bin2417	26.35	2.67	−1.35	7.3
	qGWP9.1	2017-WW	9	Bin3222	2.76	2.03	−1.24	5.5
	qGWP9.2	2017-HN	9	Bin3294	20.25	2.34	−1.48	6.4
		2018-HN	9	Bin3277	16.57	3.71	−2.43	9.9
	qGWP9.3	2018-HN	9	Bin3406	53.87	4.60	−3.25	12.2
TGW	qTGW4.1	2017-WW	4	Bin1233	18.15	2.21	−0.09	6.0
	qTGW6.1	2017-HN	6	Bin1828	127.46	2.39	0.07	6.7
	qTGW8.1	2017-WW	8	Bin2464	46.39	2.56	−0.06	6.9
	qTGW8.2	2017-WW	8	Bin2608	107.93	2.35	−0.06	6.4

+ and−: Positive values indicate that the Longgu7 allele increased the trait value and negative values indicate that the Yugu1 allele increased the trait value. PWE is abbreviation for phenotypic variance explained. Traits were straw weight per plant (SWP), panicle weight per plant (PWP), grain weight per plant (GWP), and 1000-grain weight (TGW). Environments were Dunhuang (DH), Huining (HN), Wuwei (WW) and Gangu (GG). 2017 and 2018 represented years

#### QTL of straw weight per plant

Seventeen QTLs of straw weight per plant were identified on Chr1, Chr2, Chr3, Chr6, Chr7, Chr8 and Chr9 and explained 5.6–14.7% of the phenotypic variation (Table 4). Of them, qSWP7.4 and qSWP9.1 were detected across multi-environments and favorable alleles came from Yugu1. Four QTLs including qSWP2.1, qSWP6.2, qSWP7.1 and qSWP8.1 were identified under two environments and favorable alleles were derived from Yugu1 except qSWP6.2. Remaining 11 QTLs were only detected in a single environment, and favorable alleles came from Yugu1 except favorable alleles of qSWPL6.1 and qSWP8.2 from Longgu7.

#### QTL of panicle weight per plant

Fourteen QTLs for panicle weight per plant were mapped on Chr2, Chr3 Chr5, Chr6, Chr7, Chr8 and Chr9, and explained 5.5–10.9% of the phenotypic variation (Table 4). Among these QTLs, qPWP3.2, qPWP3.3, qPWP6.3 and qPWP9.2 were mapped under two environments, and favorable alleles originated from Yugu1 except qPWP6.3. Other QTLs of PWP were detected in a single environment and the effects for these QTLs except qPWP6.2 were from Yugu1 alleles.

#### QTL of grain weight per plant

Twelve QTLs for grain weight per plant were mapped on seven chromosomes, explaining 5.5–12.2% of the phenotypic variance (Table 4). Chr2, Chr3, Chr6, Chr7, Chr8 and Chr9 contained 2, 3, 1, 2, 1 and 3 QTLs, respectively. Among these QTLs, qGWP3.3 was identified crossing three environments and favorable alleles for increasing the trait value came from Yugu1. Furthermore, qGWP3.1, qGWP3.2 and qGWP9.2 from Yugu1 and qGWP6.1 from Longgu7 were detected in two environments, whereas the rest QTLs were detected in a single environment and favorable alleles for increasing the trait value were derived from Yugu1 except qGWP2.1.

#### QTL of 1000-grain weight

Four QTLs for 1000-grain weight were identified on Chr4, Chr6 and Chr8, which explained 6.0–6.9% of the phenotypic variance (Table 4). Three QTLs, named qTGW4.1, qTGW8.1 and qTGW8.2, were detected in 2017 WW environment, and favorable alleles for increasing the trait value came from Yugu1. Another QTL was mapped on Chr6 in a single environment and favorable allele was derived from Longgu7.

### Stable QTL and QTL clusters

**Fig. 3:**
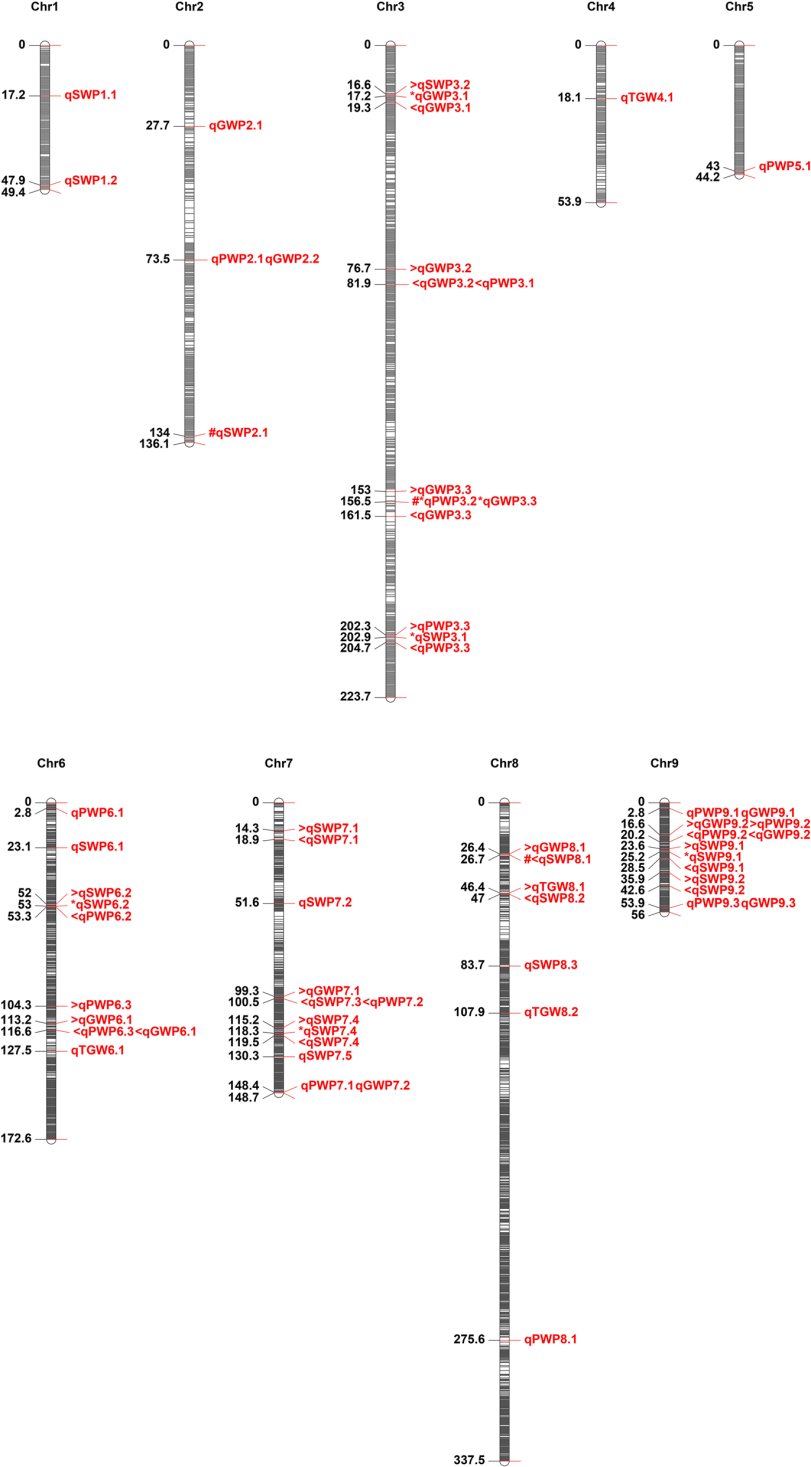
QTL controlling yield component traits on nine chromosomes. The color intensity of the bar chart represents the marker density. The number on the left indicates the genetic distance in centimorgan (cM). On each chromosome, the name of each QTL is shown on the right. Parallel QTLs indicate the same location on the chromosome. The symbol’<, *, >’ in front of the QTL represent partial overlap with the QTL above, the both flanking QTL and the QTL below region, respectively. The symbol’#’ in front of the QTL represents the same QTL identified under two environments. QTL were identified for four yield traits and shown as straw weight per plant (SWP), panicle weight per plant (PWP), grain weight per plant (GWP), and 1000-grain weight (TGW)

Three QTLs named qGWP3.3, qSWP7.4 and qSWP9.1 were detected in all three environments (Table 4, Fig. 3). Among them, qGWP3.3 was mapped between Bin0982 and Bin1009 spanning physical interval of 87.41 kb. qSWP7.4 was between Bin2250 and Bin2263 covering genomic region for 415.94 kb, and qSWP9.1 was located on the physical interval between position 24,283,629 and 29,391,213 on Chr9. Then, we searched for the genes within the mapping regions of three QTLs at Phytozome (https://phytozome.jgi.doe.gov/pz/portal.html). Seven, 42 and 76 genes were identified in the mapping interval for qGWP3.3, qSWP7.4 and qSWP9.1, respectively (Additional file 6: Table S6). QTL clusters were defined as a chromosome region which contained multiple QTLs for various traits within ~ 20 cM [28]. In this study, nine QTL clusters were found on chromosome 3, 6, 7 and 9 (Fig. 3). Among these, Chr3 harbored four QTL clusters, including a stable qGWP3.3. Chr6 and Chr7 had the two clusters, one of which on Chr7 contained the stable qSWP7.4. Chr9 carried one QTL cluster for SWP, PWP, and GWP and contained the stable qSWP9.1. Interestingly, all favorable alleles of QTL clusters on Chr6 for SWP, PWP, GWP and TGW origin from Longgu7, whereas, all favorable alleles of QTL clusters on Chr3, Chr7 and Chr9 were from Yugu1 except TGW.

## Discussion

### A novel high-density linkage map

Genetic linkage map is the basis for QTL mapping and gene cloning. Its application value depends on the number of markers, the saturation of the map, and the uniformity of the distribution of markers on the map [25]. Therefore, a construction of a high-density linkage map could improve the accuracy of QTL mapping [27]. In recent years, with the development of sequencing technology and genome assemblies, SNP [12, 26, 27], SSR [16, 29, 30] can be massively obtained. In present study, we sequenced a RIL population using high-throughput sequencing methods and constructed a high-density genetic map with 3413 bin markers carried 1,047,978 SNPs. Compared with the previously reported bin-marker genetic maps, the genetic map spanning 1222.26 cM had higher saturation and more markers. For example, Zhang et al. [25] constructed a linkage map consisted of 2022 bin markers harboring 33,579 SNPs, covering 1934.6 cM of the genome. Wang et al. [27] developed a Bin genetic linkage map with a total of 3129 Bins from 48,790 SNPs. But the present map still has unevenly distributed markers across nine chromosomes. It may be caused by high sequence similarity in particular regions between parents. For instance, chromosomes with fewer SNPs (Chr1, Chr4, Chr5) might have low SNPs diversity between two parents. Fang et al. [16] found similar results in the linkage map with 1013 SSRs markers constructed from F_2_ population. However, the new map was constructed via RIL population with phenotypic stability, more markers (3413 bin markers), higher density (8.81 bin markers/Mb) and covered the whole genome. Thus, it can be used in better dissecting the genetic mechanism of diverse traits in foxtail millet.

### Segregation distortion

Segregation distortion is commonly recognized as a potentially powerful evolutionary force and has occurred widely in mapping populations [31, 32]. It is caused by lethality, partial male or female sterility, gametic selection or zygotic selection and/or pollen spine development [31, 33], which become more serious in RIL populations because of genetic drift [31] was associated with both natural and artificial selection for several generations [16, 34]. Zhang et al. [25] found segregation distortion on Chr6 which was significantly distorted toward Zhanggu which may exist intraspecific hybrid pollen sterility, and they located one gene controlling the high male-sterility QTL combined with previous report [35]. Similarly, Fang et al. [16] found two gametocidal genes (Gc) on Chr8 by the distorted loci in two SDRs skewed toward different parents. In the present study, there were two identical SDRs at the middle-upper and bottom on Chr8, suggesting that the two distorted loci were immobilized in F_2_ and RIL populations constructed from Longgu7 × Yugu1. In addition, the present study exhibited more general segregation distortion (*p* < *0.05*) accounting for 89.10% of the total bin markers, with 350 (11.93%) bin markers attributing to Yugu1 alleles and the remaining bin markers (88.07%) favoring Longgu7 alleles. We found that no matter in F_2_ or RIL, segregation distortion was toward to Longgu7 which was bred by our research group for many years at HN where our RIL population was also constructed. And recombinant fraction of markers on all SDRs was lower than other regions. Taking these results together, we deduced that was closely related to the accumulation of natural selection effect and the tighter linkage of chromosome fragment on SDRs as the number of self-crossing generation increases.

### QTL regions for yield component traits

Straw weight per plant, panicle weight per plant, grain weight per plant and 1000 grain weight are the main yield component traits of foxtail millet. Construction of a high-density linkage map laid a foundation for the accuracy of QTL mapping for these yield traits. In present study, a total of 47 QTLs on 9 chromosomes for four yield component traits were detected. Among these, three stable QTLs, namely qGWP3.3, qSWP7.4 and qSWP9.1 identified across the multi-environments will be the value information for breeding improvement of yield component traits. qGWP3.3 is different from either of reported TGW QTLs at position 1,472,987–1,504,380 by Zhang et al. [25] and position 7,027,285–7,177,203 by Wang et al. [27] on the same chromosome 3. This suggests that qGWP3.3 might be new and major loci that was associated with grain weight of foxtail millet. Of course, the different QTLs may result from gene by environment interaction. The stable qSWP7.4 identified for SWP in the study was located on the physical interval between position 18,175,731 and 18,591,672 on Ch7, which was adjacent to the locus near GSA07381a (19397488) identified by Fang et al. [16] using F_2_ population from a cross between the same biparents, indicating a robust QTL for SWP. qSWP9.1 (24,283,629–29,391,213) on Chr9 was overlapped with two reported SNP loci for tiller number and total panicles number per plant (23,096,040; 32,059,125) detected by Jia et al. [23]. Seven, 42 and 76 genes in the intervals of qGWP3.3, qSWP7.4 and qSWP9.1 were identified according to the gene annotation at Phytozome (https://phytozome.jgi.doe.gov/pz/portal.html). And some of genes, such as *Seita.7G078300* and *Seita.9G275800* which were homologous to *OsFBL16* [36, 37] and *LOC_Os10g20260* [38] that are related to plant growth and development and grain beta-glucan (BG) synthesis in rice, were likely to be candidate genes. But the functions of these genes were still unknown in foxtail millet. In addition, the nine QTL clusters on Chr3 (6,565,090–7,781,600; 17,115,096–39,392,422; 44,312,207–44,329,955; 46,413,267–46,599,898), Chr6 (3,256,245–3,528,127; 6,659,067–7,006,735), Chr7 (13,552,620–13,884,797; 18,175,731–20,680,906) and Chr9 (9,022,723–20,276,901) could be associated with the complex relationship among yield traits [27]. Thus, they may be involved pleiotropic genes or closely linked alleles [16]. Furthermore, all favorable alleles of QTL cluster on Chr6 originated from Longgu7, which yield component traits were lower than those in Yugu 1, suggesting that the parent with low phenotypic values also carried favorable alleles for boosting yield component traits. Taken together, these stable and QTL clusters laid a foundation for fine mapping, identifying candidate genes, elaborating molecular mechanisms and application in foxtail millet molecular breeding.

## Conclusions

In present study, a high-density genetic map including 3413 bin markers was constructed, which covered 1222.26 cM with an average distance of 0.36 cM between consecutive bin markers. Three stable QTLs and nine QTL clusters on the chromosome 3, 6, 7, 8 and 9 were identified, which could be applied preferentially for fine mapping, candidate genes identification and application in foxtail millet breeding programs by marker-assisted selection.

## Methods

### Plant materials and phenotyping

Longgu 7, a cultivar from spring sowing region in northwest China, which has shorter growth duration, lower plant height, lower biomass and grain yield per plant, was selected as the male parent line and Yugu1, a cultivar from summer sowing region in north of central China, which has longer growth duration, higher plant height, higher biomass and grain yield per plant, was used as the female parent line. Hybridization was performed between Longgu7 and Yugu1, and F_1_ individuals were obtained in winter of 2012 in Sanya. During spring 2013 in Sanya, F_1_ seeds was sown and self-pollinated to produce the F_2_ individuals. One hundred and sixty-four F_2:8_ RILs were obtained using a single seed descent strategy in Huining, Gansu, China. The F_2:8_ RILs along with parents were grown three different environments in Dunhuang (DH, coordinates: 94.65°E/40.17°N), Huining (HN, coordinates: 105.09°E/35.56°N) and Wuwei (WW, coordinates:102.48°E/37.92°N) in 2017. F_2:9_ segregation population and parents were grown two different environments in Gangu (GG, coordinates: 105.33°E/34.79°N) and Huining mentioned above in 2018. Among these test environments, DH and WW belong to irrigated agricultural areas, while HN and GG were rain-fed agricultural areas in northwest China. SWP, PWP, GWP and TGW of Longgu7 were all lower than Yugu1 on all test environments. Fresh leaf tissues of the parents and 164 F_2:8_ RILs planted in HN were sampled for sequencing during jointing stage. After ripening, 15 plants of the two parents and 164 RILs under all test environments were randomly selected in the field and the yield component traits including SWP, PWP, GWP and TGW were measured by electronic balance with accuracy for 0.01 g. SPSS Statistics 17.0 was used to perform descriptive statistics, correlation and univariate general linear model analyses for yield component traits.

### Sequencing of the parental lines and RIL population

Young leaf tissues of two parental lines and 164 F_2:8_ RILs samples were used to extract total genomic DNA with the CTAB method [16]. DNA degradation and contamination of all lines were monitored on 1% agarose gels. The NanoPhotometer® spectrophotometer (IMPLEN, CA, USA) and Qubit® DNA Assay Kit in Qubit® 2.0 Flurometer (Life Technologies, CA, USA) were used to check and measure DNA purity and concentration, respectively. A total amount of 1.5 μg DNA per sample were used as input material for the DNA sample preparations. Sequencing libraries were generated using Truseq Nano DNA HT Sample preparation Kit (Illumina USA) and index codes were added to attribute sequences to each sample. The libraries constructed were sequenced by Illumina HiSeq platform and 150 bp paired-end reads were generated with insert size around 350 bp. Reads with ≥10% unidentified nucleotides, > 50% bases having Phred quality < 5, > 10 nt aligned to the adapter and putative duplicated reads were removed and the remaining high-quality clean reads were used in SNP calling.

### Sequence alignment, genotyping, and recombination breakpoint determination

The reference genome sequence of *Setaria italica* downloaded from Phytozome (*Setaria*_*italica*_v2.0) was used as a reference to align with reads of the parents and 164 RILs by BWA software (Ver. 0.7.17,) [39]. SNPs from alignment between parents and reference genome were flited out to generate specific SNPs with SAMtools (Ver. 0.1.8,) [40] and BCFtools (Ver. 1.3.1) [41]. The specific SNPs positions were marked for RIL SNP calling. The genotype of RILs was converted to 1 if the SNP was the same as Longgu7, else the genotype of RILs was converted to 0. Bin markers were obtained from all lines by sliding 15 SNPs as the window with R script. Based on the highest probability of a genotype, the sum of 15 SNPs was greater than 10.5 that was considered from Longgu7, and less than 10.5 that was considered from Yugu1 [42]. The obtained bin markers were used to detect recombination breakpoint on chromosome by customized PERL scripts, where it appeared between two different bin markers.

### Genetic map construction and QTL mapping

R package ‘onemap’ and ‘Linkagemapveiw’ were used to analyze linkage distance and construct the linkage map, respectively. MapQTL 6.0 was applied to detect QTL by Multiple QTL mapping. A threshold of log of odds (LOD) ≥ 2.0 indicated the existence of QTL [31]. Positive additive effects indicated that alleles originating from Longgu7 increased the phenotypic value, while negative additive effects indicated that alleles derived from Yugu1 increased the phenotypic value. QTL with partially or fully overlapping confidence intervals was regarded as the same QTL. The QTL nomenclature was designated beginning with a letter “q”, followed by the trait abbreviation as mentioned above, the chromosome number and the QTL serial number.

## Supplementary information

**Additional file 1: Table S1.** Number of genes, SNPs, InDels and specific SNPs on nine chromosomes of the two parents by aligning against. reference genome.

**Additional file 2: Table S2.** Bin markers and genotypes of 164 RILs.

**Additional file 3: Table S3.** Markers distribution and chromosome parameters on the linkage map.

**Additional file 4: Table S4.** Recombinant breakpoints and corresponding physical locations.

**Additional file 5: Table S5.** The chi-squared tests of bin marker of RIL population.

**Additional file 6: Table S6.** The number of gene within physical interval of qGWP3.3, qSWP7.4 and qSWP9.1.

**Additional file 7: Figure S1.** A high-density linkage map based on resequencing a RIL population in foxtail millet.

**Additional file 8: Figure S2.** Marker and interval profiles of segregation distortion and estimated recombination fraction for RIL.

## Data Availability

Raw sequencing data related to this study has been deposited at NCBI under an SRA accession number PRJNA562988 and can be accessed through the link https://www.ncbi.nlm.nih.gov/bioproject/PRJNA562988.

## Abbreviations

Chr: Chromosome
GWAS: Genome wide association studies
GWP: Grain weight per plant
LOD: Log of odds ratio
MAS: Marker assisted selection
PVE: Phenotypic variance explained
PWP: Panicle weight per plant
QTL: Quantitative trait locus/loci
RAD-seq: Restriction site-associated DNA sequencing
RIL: Recombinant inbred line
SNP: Single nucleotide polymorphism
SSR: Simple sequence repeat(s)
SWP: Straw weight per plant
TGW: 1000-grain weight
