# Supplementary material for: QTL mapping of yield component traits on bin map generated from resequencing a RIL population of foxtail millet (Setaria italica)
Source: BMC Genomics. 2020 Feb 10;21:141. doi: 10.1186/s12864-020-6553-9 (PMC7011527; doi:10.1186/s12864-020-6553-9)
Supplement: Supplementary file 1 — Additional file 1: Table S1. Number of genes, SNPs, InDels and specific SNPs on nine chromosomes of the two parents by aligning against. reference genome. [file 12864_2020_6553_MOESM1_ESM.doc]

**Table S1** Number of genes, SNPs, InDels and specific SNPs on 9 chromosomes by the two parents by aligning against reference genome.

| Chromosome | Forward gene | Reverse gene | ^a^SNP | ^a^Indel | ^b^SNP | ^b^Indel | ^a^Specific SNP | ^b^Specific SNP |
| --- | --- | --- | --- | --- | --- | --- | --- | --- |
| Chr1 | 2047 | 1954 | 131382 | 13168 | 90788 | 7843 | 50259 | 9665 |
| Chr2 | 2378 | 2216 | 252914 | 21254 | 157896 | 11862 | 124394 | 29376 |
| Chr3 | 2095 | 2157 | 219464 | 20789 | 212987 | 17784 | 88276 | 81799 |
| Chr4 | 1486 | 1562 | 102486 | 9819 | 76687 | 6200 | 36140 | 10341 |
| Chr5 | 2517 | 2380 | 169464 | 16684 | 143909 | 11776 | 55186 | 29631 |
| Chr6 | 1322 | 1284 | 158455 | 14625 | 110953 | 8823 | 70932 | 23430 |
| Chr7 | 1775 | 1691 | 222780 | 18642 | 138565 | 9464 | 100224 | 16009 |
| Chr8 | 1354 | 1224 | 379890 | 27044 | 293496 | 19078 | 149341 | 62947 |
| Chr9 | 2996 | 3080 | 228334 | 19577 | 169380 | 10879 | 84491 | 25537 |
| Total | 17970 | 17548 | 1865169 | 161602 | 1394661 | 103709 | 759243 | 288735 |

^a^ represents Longgu 7, ^b^ represents Yugu1.
