# Supplementary material for: QTL mapping of yield component traits on bin map generated from resequencing a RIL population of foxtail millet (Setaria italica)
Source: BMC Genomics. 2020 Feb 10;21:141. doi: 10.1186/s12864-020-6553-9 (PMC7011527; doi:10.1186/s12864-020-6553-9)
Supplement: Supplementary file 3 — Additional file 3: Table S3. Markers distribution and chromosome parameters on the linkage map. [file 12864_2020_6553_MOESM3_ESM.docx]

**Table S3** Markers distribution and chromosome parameters on the linkage map

| **Chromosome** | **Bin markers** | **Break points** | **Distortion loci** | **Distortion rate**  **(%)** | **SDR** | **Linkage length**  **(cM)** | **Average interval**  **(cM)** | **Physical length**  **(Mb)** | **Marker density**  **(marker/Mb)** |
| --- | --- | --- | --- | --- | --- | --- | --- | --- | --- |
| Chr1 | 184 | 161 | 184 | 100.0 | 1 | 49.43 | 0.27 | 42.15 | 3.82 |
| Chr2 | 347 | 441 | 311 | 89.63 | 2 | 136.14 | 0.39 | 49.20 | 7.05 |
| Chr3 | 647 | 726 | 559 | 86.40 | 6 | 223.75 | 0.35 | 50.65 | 12.77 |
| Chr4 | 154 | 175 | 124 | 80.52 | 1 | 53.87 | 0.35 | 40.41 | 3.81 |
| Chr5 | 163 | 144 | 163 | 100.0 | 1 | 44.21 | 0.27 | 47.25 | 3.45 |
| Chr6 | 491 | 561 | 425 | 86.56 | 5 | 172.63 | 0.35 | 36.01 | 13.64 |
| Chr7 | 374 | 482 | 301 | 80.48 | 5 | 148.69 | 0.40 | 35.96 | 10.40 |
| Chr8 | 852 | 1094 | 667 | 78.29 | 9 | 337.52 | 0.40 | 40.69 | 20.94 |
| Chr9 | 201 | 179 | 201 | 100.0 | 1 | 56.02 | 0.28 | 58.97 | 3.41 |
| Total | 3413 | 3963 | 2935 | 89.10 | 31 | 1222.26 | 0.34 | 401.29 | 8.81 |

SDR: segregation distorted region
