# Supplementary figures and images for: QTL mapping of yield component traits on bin map generated from resequencing a RIL population of foxtail millet (Setaria italica)
Source: BMC Genomics. 2020 Feb 10;21:141. doi: 10.1186/s12864-020-6553-9 (PMC7011527; doi:10.1186/s12864-020-6553-9)

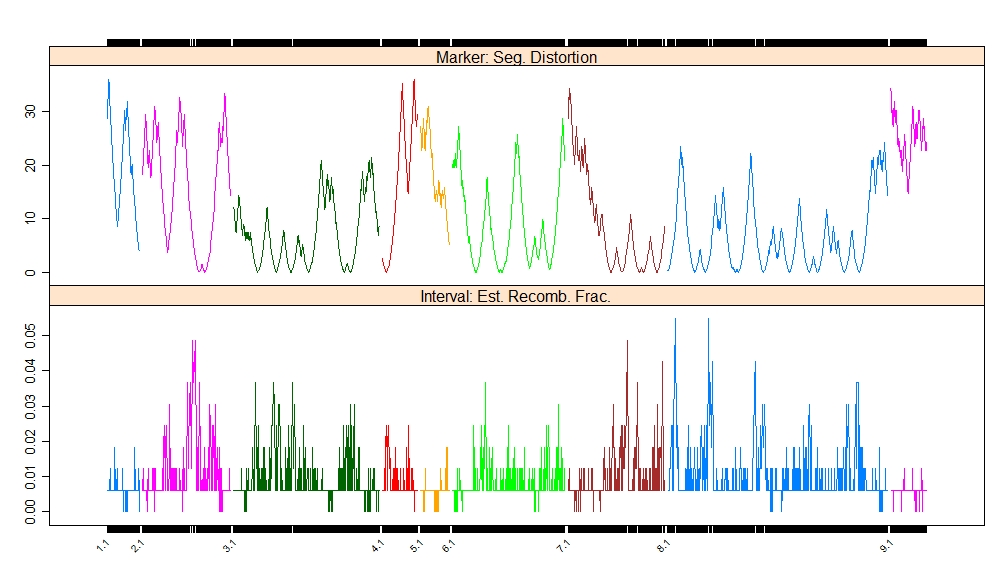

Supplement: Supplementary file 8 — Additional file 8: Figure S2. Marker and interval profiles of segregation distortion and estimated recombination fraction for RIL. [file 12864_2020_6553_MOESM8_ESM.jpeg]
