# Supplementary material for: QTL mapping of yield component traits on bin map generated from resequencing a RIL population of foxtail millet (Setaria italica)
Source: BMC Genomics. 2020 Feb 10;21:141. doi: 10.1186/s12864-020-6553-9 (PMC7011527; doi:10.1186/s12864-020-6553-9)
Supplement: Supplementary file 7 — Additional file 7: Figure S1. A high-density linkage map based on resequencing a RIL population in foxtail millet. [file 12864_2020_6553_MOESM7_ESM.pdf]

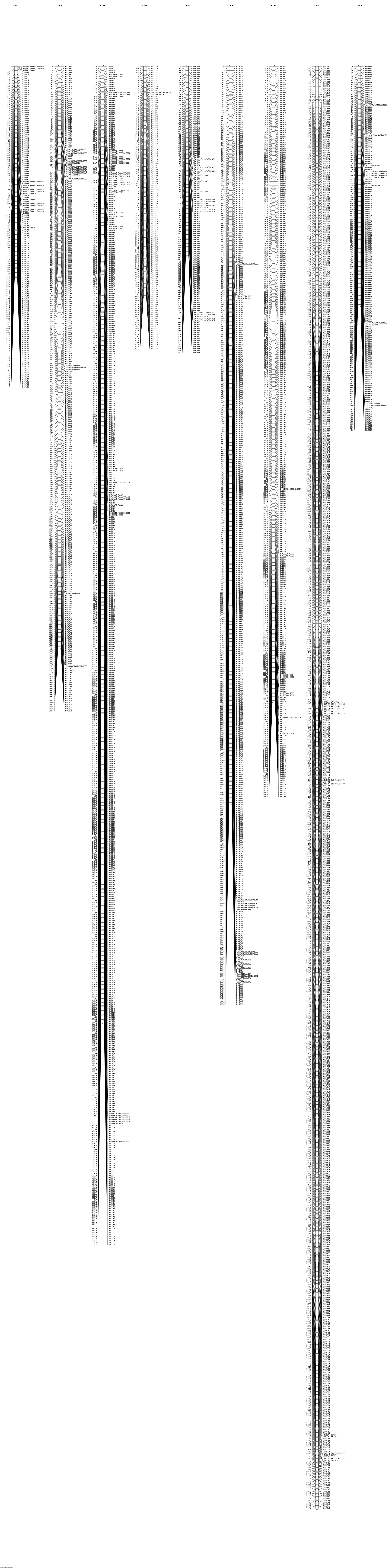
Chr1
Chr2
Chr3
Chr4
Chr5
Chr6
Chr7
Chr8
Chr9
